# *Anoplophora glabripennis*, an invasive longhorned beetle, has the potential to damage fruit trees in Japan

**DOI:** 10.1038/s41598-024-63548-0

**Published:** 2024-06-03

**Authors:** Hiroe Yasui, Nao Fujiwara-Tsujii, Soichi Kugimiya, Kazuki Shibuya, Koji Mishiro, Nami Uechi

**Affiliations:** Institute for Plant Protection, National Agriculture and Food Research Organization, Tsukuba, Ibaraki 305-8666 Japan

**Keywords:** *Anoplophora glabripennis*, ALB, Trees, Orientation, Feeding preference, Oviposition preference, Entomology, Invasive species

## Abstract

Invasive *Anoplophora glabripennis* recently became established in Japan and has caused heavy damage to several street-tree species. Overseas, *A. glabripennis* infests trees of the genera *Acer* and *Populus* as common host plants, and *Malus*, *Pyrus*, and *Prunus* (Rosaceae), including apple, pear, and plum trees; it therefore poses a potential risk to the production of economically valuable fruits in Japan. Fruit farms in areas already invaded by *A. glabripennis* are now threatened with tree infestation. We aimed to determine the potential damage to major fruit species in Japan. In the laboratory, we determined if the adult beetle is attracted to the odor of each of these tree species’ branches; two confirmed host plant species and five Rosaceae fruit species, as well as its feeding preferences among branches of one host plant and the five fruit trees and its oviposition preferences among them. Among the fruit species, cherry branch had the highest rate of odor orientation by males. The feeding-preference assay showed that, besides the host plant, Japanese pear was the most consumed among the fruit trees. The potential risk of *A. glabripennis* laying eggs on fruit-tree branches was high for Japanese pear and above zero for plum, apple, and cherry branches.

## Introduction

The Asian longhorned beetle (ALB) *Anoplophora glabripennis* (Motschulsky) (Coleoptera: Cerambycidae) originated in China and Korea^1^. It has already invaded and become established in some European countries (Italy, Switzerland, France, and Germany) and several US states. In these countries, there are ongoing efforts to eradicate the beetle^2,3^. It has been eradicated in Austria, Canada, Finland, and the United Kingdom^2^. It was initially reported in Japan as being locally and temporarily present, but since 2021 it has become established in 13 prefectures^4–16^.

In Japan, *A. glabripennis* has caused substantial damage, mainly to street trees such as *Cercidiphyllum japonicum* (*katsura* in Japanese), *Ulmus parvifolia* (Chinese elm), *Aesculus turbinata* (Japanese horse chestnut), and *Salix* spp. (willow)^4–16^. *Cercidiphyllum japonicum* is distributed all over Japan, as well as in China and on the Korean Peninsula, and it has been planted in the United States and other countries. During their development, the larvae of *A. glabripennis* damage tree trunks and branches by boring inside them, often causing death of the host trees. Trees mainly planted in Europe and China were categorized into four groups by van der Gaag and Loomans^17^ based on the life cycle completion of *A. glabripennis*, and all four of these host-plant species that have been damaged by *A. glabripennis* in Japan have been categorized into the first of four categories, namely “Category I: preferred plants,” which are those on which *A. glabripennis* has completed its life cycle under field conditions^17,18^. In the US, the trees are also categorized into four groups according to the degree of damage caused by *A. glabripennis*, namely “preferred host,” “occasional to rare host,” “questionable host” (including no records from China), and “no US record” (records from China but not in US)^19^. *Cercidiphyllum japonicum* is categorized as an “occasional to rare host” in the US. The difference between the US and Europe-China categorization suggests that the plant species preferred by *A. glabripennis* may vary by region.

The potential risk posed by *A. glabripennis* to the production of economically valuable fruit has been highlighted by the substantial damage caused to stone fruit trees in Japan by the invasive longhorned beetle *Aromia bungii* (Coleoptera: Cerambycidae) in the last 5 years^20–23^. Overseas, *A. glabripennis* infests trees belonging to the genera *Acer* and *Populus* as common host plants, and *Prunus*, *Pyrus*, and *Malus* (Rosaceae), including commercially important deciduous fruit trees such as plums, pears, and apples^1–3,18^. Among fruit trees, *Malus* is categorized as Category I: completion of the beetle’s life cycle has been confirmed on two living trees of this genus in China, and also in a caged test in Austria^2,17,18^. In *Pyrus*, only Chinese pear (*Pyrus bretschneideri*) is categorized as Category I; in other words, *A. glabripennis* has completed its life cycle in this plant species in the field. Other *Pyrus* species are categorized as Category IV “others”. There are no reports of the occurrence of the beetle on Japanese pear (*Pyrus pyrifolia*) cultivated in Japan. Moreover, there are no confirmed reports of life-cycle completion in the genus *Prunus*, which includes important Japanese fruits (peach, plum, and oriental plum). Only two (non-fruit) species of *Pyrus* have been placed in Category III (“plant species on which *A. glabripennis* has been reported to complete part of its life cycle”)^2,17^. In the US, *Malus*, *Prunus*, and *Pyrus* are listed under “questionable US records,” so it is still possible that the beetle can damage these trees^19,24^.

The above studies indicate that the details of *A. glabripennis* infestation are uncertain in Japan. We therefore aimed to clarify the potential damage to major deciduous fruit species in this country. Here, in a laboratory study, we determined if the adult beetle is attracted to the volatiles of each of these tree species’ branches; two host plants (*C. japonicum* and *A. turbinata*) and five fruit plants (apple: *Malus pumila*; Japanese pear: *Py. pyrifolia*; peach: *Prunus persica*; Japanese plum: *Prunus mume*; and cherry: *Prunus avium*). We also evaluated the beetle’s feeding preference among the branches of one host plant (*C. japonicum*) and the above mentioned five fruit trees, and the oviposition preference of females among them. Farmers in fruit-producing prefectures where *A. glabripennis* has already become distributed are concerned about damage to their fruit trees. Peach, apple, and Japanese pear are particularly important Japanese fruits^25^ with the potential to be damaged by *A. glabripennis*. If the beetle is found to be damaging these fruit trees, Japanese fruit production could suffer very serious damage. Therefore, we also designed a feeding-choice bioassay by modeling a situation in which the fruit-tree orchards neighbored host trees, namely a choice bioassay between the branches of the two host plants (*C. japonicum* and *A. turbinata*) and three fruit trees (*M. pumila*, *Py. pyrifolia*, and *Pr. persica*), to estimate the potential for damage to these fruit trees.

## Results

### Orientation of adult beetles to volatiles from host and fruit-tree branches

**Figure 1:**
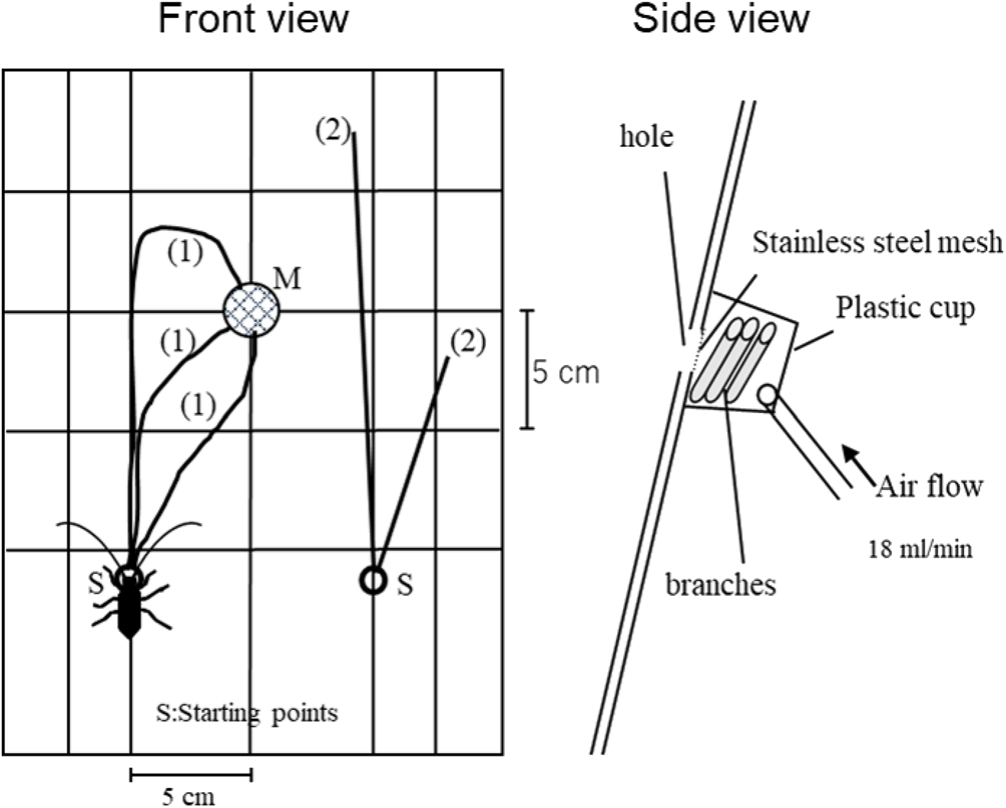
Arena used to assay the orientation of individual adult *Anoplophora glabripennis* beetles to volatiles from cut branches. Males or females were introduced individually at the right or left starting points (S). (1) When the adult veered or curved to walk towards the hole (M) within two min, this was considered a positive response; (2) when the adult walked straight up, this was considered a negative response.

**Figure 2:**
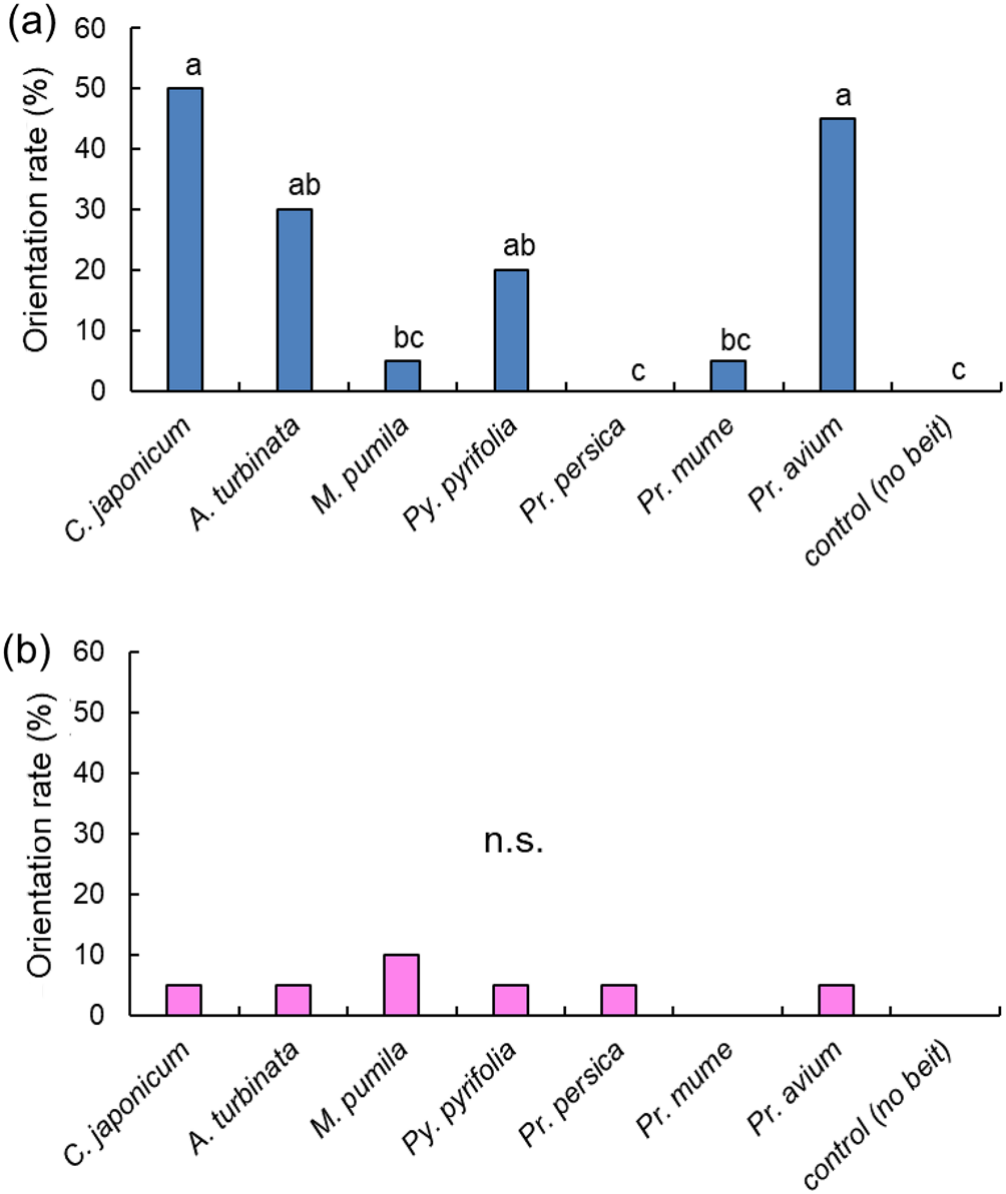
Rates of orientation of adult *Anoplophora glabripennis* beetles to volatiles from branches of host plants and fruit trees. (**a**) Males, (**b**) Females. Assays of *A. turbinata* and *Pr. avium* were performed in 2023; others were done in 2022. N = 20 for each sex.

The behavior assay described by Fukaya et al.^26^ was modified to examine the response of* Anoplophora glabripennis* adults to volatiles released by branches (Fig. 1). Their orientation responses to the various tree branches differed significantly (chi-square test; *χ*^2^ = 36.8, *df* = 7, *P* < 0.001; Fig. 2). Over 45% of males showed a positive response (i.e., movement toward the stimulus) to volatile chemicals from the host plant *Cercidiphyllum japonicum* (Fig. 2). These values for the host plants *C. japonicum* and *Aesculus turbinata* (30%) were significantly higher than that for the unbaited control (Ryan’s test; *P* < 0.05). Surprisingly, the males’ responses to *Prunus avium* (45%) and *Pyrus pyrifolia* (20%) did not differ significantly from their responses to the host plant positive controls (*P* > 0.05). Fewer than 5% of males showed an orientation response to volatiles from each of the remaining fruit trees. Females showed low positive orientation responses to the volatiles from all of the host-plant or fruit-tree branches under these assay conditions (*χ*^2^ = 3.44, *df* = 7, *P* = 0.84).

### Assay of consumption by adults among one host and five fruit-tree branches

**Figure 3:**
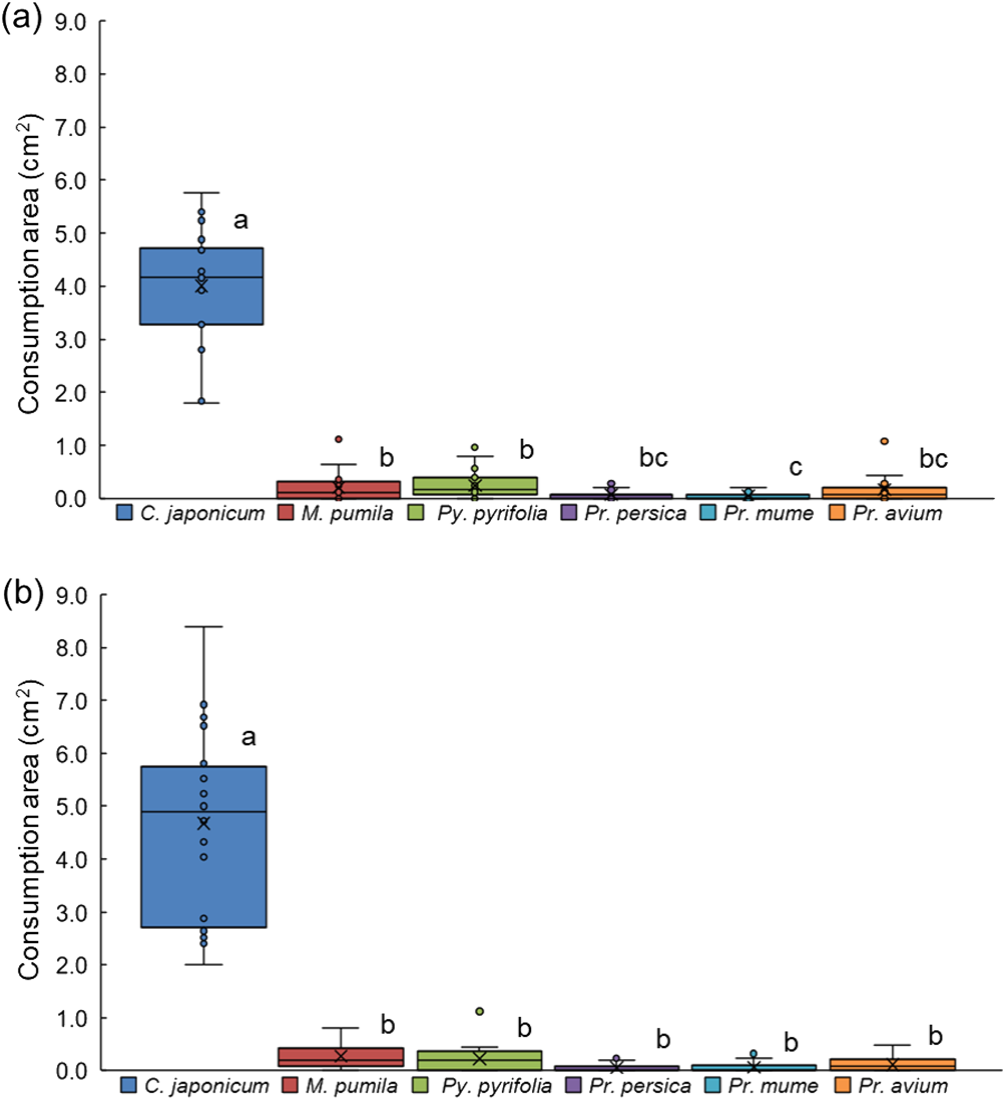
Feeding-choice assay of adult *Anoplophora glabripennis* beetles among the branches of one host-plant species and five fruit-tree species. (**a**) Area consumed by each male, (**b**) area consumed by each female for 24 h-duration. Box plots: middle line, mean; boxes, quartiles; whiskers, maximum and minimum values. ○ Data points; × average. Males: N = 19; females: N = 20.

In the feeding-choice bioassay, when a set of thin branches from one host plant (*C. japonicum*) and the five fruit trees was simultaneously presented to beetles of each sex (Suppl. Fig. 1a), the areas of damage differed significantly among the six branches (Friedman test; corrected *Q* = 55.8, *df* = 5, *P* < 0.001 for males; corrected *Q* = 58.2, *df* = 5, *P* < 0.001 for females; Fig. 3a,b). *C. japonicum* was significantly preferred over all the others by both males and females (Fig. 3a,b; Suppl. Fig. 2a,b). Among the five fruit-tree branches, that of *Prunus mume* was the least consumed by the males (Wilcoxon signed-rank test with Bonferroni correction, *P* < 0.05/15; Fig. 3a). Females did not cause significantly different degrees of damage among the different fruit tree branches (Fig. 3b).

### Oviposition-choice bioassay among one host and five fruit-tree branches

**Figure 4:**
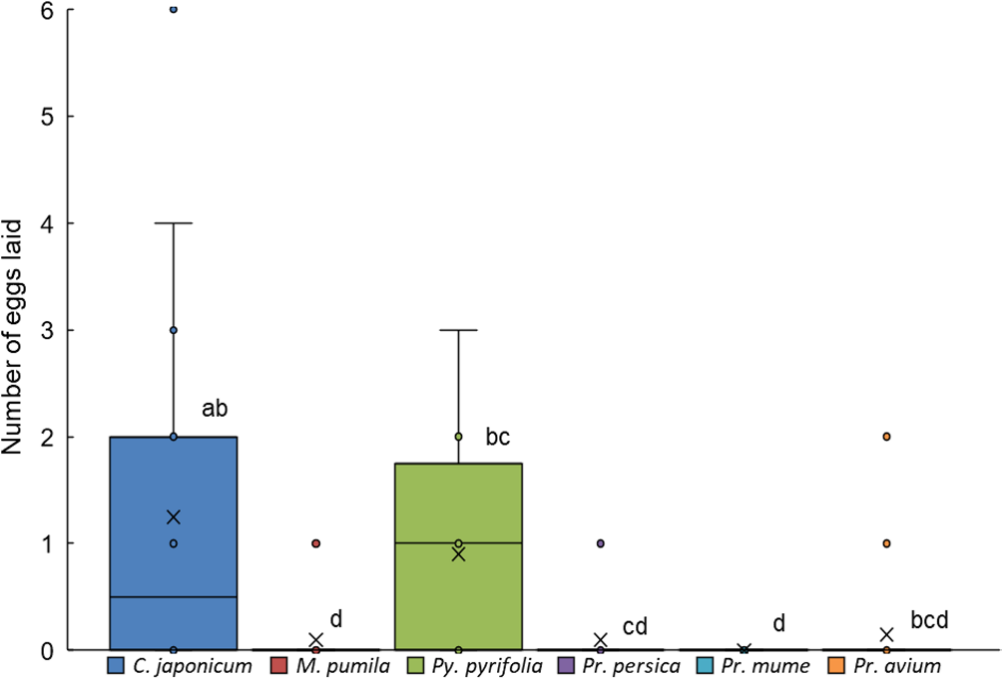
Oviposition-choice assay of female *Anoplophora glabripennis* among the branches of one host-plant species and five fruit-tree species. The number of eggs laid on each branch by each female in 2 days is shown. Box plots: middle line, mean; boxes, quartiles; whiskers, maximum and minimum values. ○ Data points; × average. N = 20.

When a set of thick branches (~ 2 cm in diameter) of the same tree species as above was presented (Suppl. Fig. 1b), females laid significantly different numbers of eggs among the six branches (Friedman test; corrected *Q* = 38.2, *df* = 5, *P* < 0.001; Fig. 4). Significantly more eggs were laid on *C. japonicum* branches than on *Malus pumila*, *Prunus persica*, and *Pr. mume* branches during the 2-day assay (Wilcoxon signed-rank test with Bonferroni correction, *P* < 0.05/15). The number of eggs laid on *Py*. *pyrifolia* branches (Suppl Fig. 1c) did not differ significantly from that on *C. japonicum* branches (Fig. 4). No eggs were found on *Pr. mume* branches. There were also large differences among replications in the numbers of eggs laid by females on the branches. The females fed mainly on the thin *C. japonicum* twigs (under 0.5 cm in diameter) provided as their food. They bit on the thick branches not for feeding but to lay their eggs under the bite sites.

### Assay of consumption by adult beetles among two host and three fruit-tree branches

**Figure 5:**
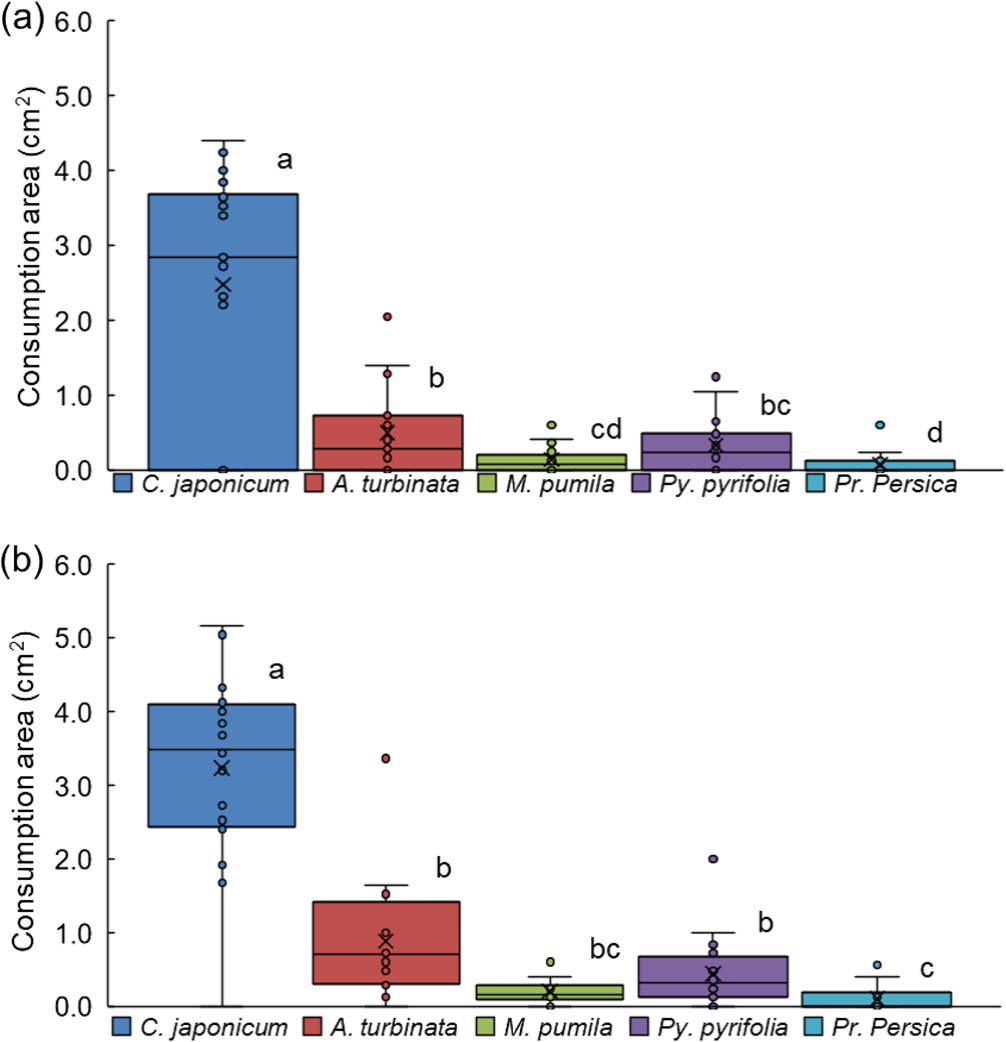
Feeding-choice assay of adult *Anoplophora glabripennis* beetles among the branches of two host-plant species and three fruit-tree species. (**a**) Area consumed by each male, (**b**) area consumed by each female. Box plots: middle line, mean; boxes, quartiles; whiskers, maximum and minimum values. ○ Data points; × average. Males: N = 19; females: N = 20.

To model the situation in fruit-producing prefectures where *A. glabripennis* is already distributed, another set of thin branches from two main host plants (*C. japonicum* and *A. turbinata*) and three important fruit-tree species (*M. pumila*, *Py. pyrifolia*, and *Pr. persica*) was simultaneously subjected to feeding choice tests. The areas of damage made by both sexes differed significantly among the five tested branches (Friedman test; corrected *Q* = 24.1, *df* = 4, *P* < 0.001 for males; corrected *Q* = 49.1, *df* = 4, *P* < 0.001 for females; Fig. 5a,b; Suppl Fig. 1d). The areas of branches of the host plant *C. japonicum* consumed by both sexes were significantly larger than those of any of the other branches (Wilcoxon signed-rank test with Bonferroni correction, *P* < 0.05/10). Among the other four types of branches, a significantly larger area of the host plant *A. turbinata* than of *M. pumila* or *Pr. persica* was damaged by male beetles, and a significantly larger area of *Py. pyrifolia* than of *Pr. persica* was damaged by the males (Fig. 5a). Female beetles inflicted significantly more damage on *A. turbinata* and *Py. pyrifolia* branches than on *Pr. persica* branches (Fig. 5b). Just after the assay started, at first bite, the males and females chose *Py. pyrifolia* at about the same rate as the two host plants (Suppl. Fig. 3a,b); this differed from the results of our other test using branches of one host and five fruit trees (Suppl. Fig. 2a,b), in which both sexes chose the host at first contact over the remaining trees.

## Discussion

Our bioassays revealed the orientation, feeding, and oviposition preference trends of invasive *Anoplophora glabripennis* adults for host and fruit-tree branches. The fruit trees used are all important in Japan and, to our knowledge, have not been assessed before for their potential risk of damage by this invasive pest. The adults used in the bioassays were collected from Japanese *katsura* trees, *Cercidiphyllum japonicum*, one of the most damaged *A. glabripennis* hosts in Japan. Therefore, we considered that the adults of *A. glabripennis* that we used had a homogeneous background in terms of plant preferences.

Males were certainly attracted to the odor of the branches of their two host plants, which were their preferred food (Fig. 2a). On the other hand, their high rate of orientation to *Prunus avium* suggests that *Pr. avium* branches contain odor components in common with those of their preferred host plants. As reviewed by Xu and Teale^27^, *A. glabripennis* discriminates host trees according to their compositions of blends of terpenes emitted from the branches. The components and ratios vary greatly for a number of reasons, all of which affect the attractiveness. It has also been reported that *A. glabripennis* is attracted to the terpene blend of maple branches damaged by conspecifics, but not to the odor of mechanically damaged or healthy maple branches^28^. It is therefore not possible to generalize that only host-specific volatiles are responsible for the attraction of this species.

Females clearly did not respond to the odor of the branches of either the host trees or the fruit trees. It has been reported before that males of *A. glabripennis* are attracted to volatiles of the host tree branches, whereas the females are not attracted to them without the presence of the volatile sex pheromones of the males^27,29^. So females may need additional stimuli to orient themselves, such as volatile sex pheromones or visual information, as is the case in *Anoplophora malasiaca*^30^. Lyu et al.^31^ has also reported that visual and olfactory cues from the host plant, alone and combined with pheromone, attracted significantly more *A. glabripennis* than equivalent cues from non-host plants. In that report, however, unlike in our study, no difference was observed in response to odors between males and females. However, Lyu et al.^31^ also reported that visual cues are initially more important than olfactory cues for orientation, suggesting that the females might use both visual and olfactory cues to find their host plants. The females may use both visual and chemical information to recognize both appropriate host trees and mates; it makes sense that the females would locate their mates on suitable plants.

In our feeding-choice bioassay among one host and five fruit trees, both sexes fed mainly on the host branches of *C. japonicum*, but they also fed on small areas of the fruit tree branches, so it is impossible to say that there is no risk of damage to these fruit trees. Whereas there was a large difference in response to plant odor between males and females, there was almost no difference in feeding preference between males and females. We consider this feeding-preference bioassay to have been an important test, because considering that adults stay on the tree while feeding, a large food intake equates to a greater chance of encountering opportunities to mate, which in turn may lead to oviposition on the tree by the female^27^.

In our oviposition-choice bioassay, the lack of a significant difference between the numbers of eggs laid on branches of the host plant *C. japonicum* and Japanese pear *Pyrus pyrifolia* suggests that this fruit tree could be a comparable oviposition site (Fig. 4; Suppl. Fig. 1d). Moreover, a few individuals also laid eggs on the branches of other fruit trees (*Malus pumila*, *Prunus persica*, and *Prunus avium*) indicating that these species also face a risk as possible oviposition sites. Only *Prunus mume* branches did not appear to be at risk of receiving the eggs of *A. glabripennis*.

Furthermore, our other feeding-choice bioassay among the branches of the two hosts and three of the fruit trees was performed to model the scenario of a highly productive district where the fruit orchards (apple: *M. pumila*; Japanese pear: *Py. pyrifolia*; peach: *Pr. persica*) were adjacent to planted sites of two heavily damaged hosts (*C. japonicum* and *A. turbinata*). The area of *C. japonicum* branches consumed was, however, significantly greater than those of any other branches, including the other host plant *A. turbinata.* This result was similar to the choice bioassay among one of the branches of the host plant and five fruit trees (Figs. 3, 5). At first bite, the males and females chose *Py. pyrifolia* at about the same rate as the two host plants (Suppl. Fig. 3a,b); this differed from the results of our other test using the branches of one host and five fruit trees (Suppl. Fig. 2a,b), in which both sexes chose the host at first contact over the remaining trees. These results mean that the beetles’ feeding preference might be plastic under different environmental situations. Final food selection by the adult beetles might also depend on the plants on which they originated; this possibility is supported by the findings of previous reports (reviewed by Xu and Teale^27^)^32^.

In the case of *Pr. persica* and *Pr. mume*, both males and females responded poorly to their odors, and the adults rarely fed on them, so it is reasonable to say that the risk of damage to these two species is low. In past studies, feeding or oviposition preference was reported using only two-choice testing between host (including various species) and non-host (including various species), or among four or five host species^32,33^. To our knowledge, ours is therefore the first report of preference-choice testing between major host and fruit-tree species, designed to estimate the risk of damage to fruit species.

In Italy, a large infestation of *A. glabripennis* was discovered in 2009^34,35^. A wide-scale field survey from 2009 covering about 5600 ha of infested land revealed 1157 infested trees out of a total of 36,361 trees up to 2019^33,36,37^. From the reported data, we calculated the percentages of infestation of each host-plant genus in the area: *Cercidiphyllum* (surprisingly, 18.2%), *Aesculus* (11.6%), *Betula* (10.2%), *Ulmus* (5.4%), *Acer* (4.2%), and *Salix* (2.8%). We also calculated the percentages for other genera, namely *Prunus* (0.66%) and *Populus* (0.12%), but we were unable to do so for other fruit tree genera because they were not reported^37^. Although the percentage of infested *Prunus* was one-fourth to one-thirtieth that of the host-tree species, this result alerted us to the risk of damage to fruit trees in the area of Italy infested by *A. glabripennis*. Following on from these field data in Italy, our results for adult damage to fruit species in our feeding-choice tests and oviposition tests may reflect the environment that contains infested areas in Japan.

As our results were obtained in the laboratory, the behavior of *A. glabripennis* in the field in Japan is still unknown. Because the species has been designated as a “specified invasive alien species” in Japan^38^, opportunities for field experiments are restricted. Therefore, even though our risk assessment was performed in the laboratory, we believe that the information obtained will be important for farmers. They should be aware of invasive species in areas where host plants have been damaged, even at this stage, when no damage by *A. glabripennis* to fruit trees has yet been found. Even if *A. glabripennis* does not have a strong preference for fruit trees, the possibility of commercial damage to fruit trees remains in situations where there are many opportunities for this species to visit the trees. In this light, in a prefecture with high levels of fruit production, a ground-planted *M. pumila* tree was covered with a net and adult *A. glabripennis* were released into the net to observe their feeding and egg-laying behaviors and larval survival^39^. Many oviposition spots and frass ejection by infested larvae were observed on the *M. pumila* tree. The investigation has been underway for only a year and will continue into the future. Meanwhile, fruit tree growers in affected areas should keep a close eye on infestations by this beetle.

Here, we used a population collected from *C. japonicum*, so the preferences of other populations, such as those found on *Salix*, *Ulmus*, or *Aesculus*, might differ. We still have no information on how *A. glabripennis* spread to various host plant trees in Japan, and the preference of these populations for both host plants and fruit trees needs to be investigated further for more efficient control of this species. Especially, the early detection of infested trees by this species is the most important.

## Materials and methods

All our experimental research on plants and insects described here complied with relevant institutional, national, and international guidelines and legislation.

### Collection of insects

*Anoplophora glabripennis* adults were collected by hand from *katsura* (*Cercidiphyllum japonicum*) trees from mid-June to July in the cities of Tsukuba, Ishioka, and Omitama (2022 and 2023) and Sendai (2022 only). Beetles were reared individually in clear plastic cups (~ 11 cm diameter × 9.5 cm high) at 24 °C under a 15-h light: 9-h dark photoperiod, illuminated by fluorescent lamps. Each *A. glabripennis* beetle was fed twigs of *C. japonicum*, which were replaced every 5 days. All cut twigs were stored at 5 °C and used within 10 days.

### Plants

Branches of *C. japonicum* were obtained from the NARO (National Agriculture and Food Research Organization) property at Tsukuba. Branches of apple (*Malus pumila*), Japanese pear (*Pyrus pyrifolia*), Japanese plum (*Prunus mume*), and peach (*Prunus persica*) used for the experiments were obtained from fruit orchards at NARO, Tsukuba, and the Horticultural Research Institute, Ibaraki Agricultural Center, Kasama. Branches of *Aesculus turbinata* and cherry tree (*Prunus avium*) were sent from the Fruits Research Institute of Fukushima Prefectural Agriculture Technology Center, Fukushima.

### Behavior assay to assess orientation

The behavior assay described by Fukaya et al.^26^ was modified to examine the response of *A. glabripennis* adults to volatiles released by branches. The control was air flow in the absence of branches. A sheet of white paper (21 × 30 cm) was fixed on a plate of the same size positioned at 75° in a clear acrylic box (30 × 30 × 30 cm). To record walking trails, thin gray lines were printed on the sheet (Fig. 1, front view). A hole (1.5 cm diameter) in the plate and paper sheet at point M was covered with mesh beneath the plate. Three branches were placed in a 60-mL plastic cup (5 cm diameter × 3.2 cm high) that was fixed beneath the plate hole. The cup was connected to an air pump (MP-2N, Shibata Scientific Technology Ltd., Tokyo, Japan) via a PTFE tube (5 mm diameter × 50 cm length), and air was blown from the hole through the cup beneath the plate at 18 mL/min (Fig. 1).

Males or females were introduced individually at the right or left starting points (S); the body axis was aligned with a vertical line (Fig. 1). The beetles walked straight up when we provided no stimuli, or they did not respond to a provided stimuli ((2) on the plate in Fig. 1). When the beetle veered or curved to walk towards the hole (M) within two min, this was considered to be a positive response ((1) on the plate in Fig. 1). When the insect failed to adjust its body axis relative to the vertical line or stopped walking for more than 2 min, the trial was aborted. The assay was conducted within 40 min after the introduction of the test stimuli. All behavioral assays were conducted in the laboratory from 10:00 to 16:00 h, at 24 °C (light period: from 4:00 to 20:00 h). All individuals were tested once a day. The bioassays for all test materials except *A. turbinata* and *Pr. avium* were performed in 2022, and those for the other two species were done in 2023. All beetles used for this assay were collected from *katsura* trees; most of them were from Ibaraki prefecture, but some used in the 2022 assays were from the city of Sendai, in Miyagi prefecture. The rates of positive responses of the beetles to the test branches were analyzed by *χ*^2^ test followed by Ryan’s method for multiple comparisons of proportions^40^. The number of replicates was 20 for each sex.

### Feeding-choice bioassay

A feeding-choice bioassay of adult beetles was conducted by using a) branches from one host-plant species and all five species of fruit tree; or b) branches from two host plant species and three of the species of fruit tree. To model the potential situations in fruit-producing prefectures, the latter setup was chosen because damage by *A. glabripennis* in the prefectures has been severe on *C. japonicum* and *A. turbinata*, and the three chosen fruit-tree species are particularly economically important in Japan. All cut branches used for the feeding bioassays were 0.7 to 1.0 cm in diameter × 4 cm long, because adult beetles always feed on the thin parts of the twigs. Females rarely oviposit on such thin twigs. Adults were starved for 16 h before the bioassay. Cut branches (one from each of the test species) were fixed with adhesive tape in a circular pattern on 15-cm-diameter filter paper (Advantec Toyo Kaisha, Ltd, Tokyo, Japan) (Suppl. Fig. 1). Males or females were introduced individually at the center of the filter paper and covered with a plastic cup (12 cm in diameter × 5 cm high) to prevent escape. After the bioassay started, the branch species that the insect bit first was recorded. After 24 h the area of each branch that had been consumed was measured with a 2-mm lattice scale printed on a transparent sheet. The area of each branch consumed by the same individual was recorded. All behavioral assays were conducted in the laboratory from 10:00 h onward at 24 °C (light period: from 4:00 to 20:00 h). The areas of test sets of branches consumed were analyzed by the Friedman test^41^ followed by Wilcoxon’s signed-rank test with Bonferroni correction. The number of replicates was 20 for each sex, but one male died during the assay (male: N = 19).

### Oviposition-choice bioassay

An oviposition-choice bioassay of females was conducted by using the branches of one host-plant species and all five species of fruit tree. All cut branches used in this bioassay were 2 cm in diameter × 5 cm long—thicker than those used in the feeding bioassays, because females are able to lay their eggs in cut branches that are at least about 2 cm in diameter. Cut branches (one from each of the test species) were fixed with adhesive tape in a circular pattern on to 15-cm-diameter filter paper (Advantec Toyo Kaisha) (Suppl. Fig. 1). The setup was covered with a plastic case (22 × 15 × 4 cm high) to prevent escape. Females were introduced individually at the center of the filter paper. As food for the female, twigs of *C. japonicum* (under 0.5 cm in diameter) were served at the center of the case. After 2 days, the number of eggs laid on the branch of each species was recorded individually. All assays were conducted in the laboratory from 10:00 h onward at 24 °C (light period: from 4:00 to 20:00 h). The numbers of eggs laid on the test sets of branches were analyzed by the Friedman test followed by Wilcoxon’s signed-rank test with Bonferroni correction. The number of replicates was 20.

### Supplementary Information

Supplementary Figures.

## Data Availability

The data presented in this study are available from the corresponding author on reasonable request.
