# Supplementary material for: Anoplophora glabripennis, an invasive longhorned beetle, has the potential to damage fruit trees in Japan
Source: Sci Rep. 2024 Jun 3;14:12708. doi: 10.1038/s41598-024-63548-0 (PMC11148008; doi:10.1038/s41598-024-63548-0)
Supplement: Supplementary file 1 — Supplementary Figures. [file 41598_2024_63548_MOESM1_ESM.pdf]

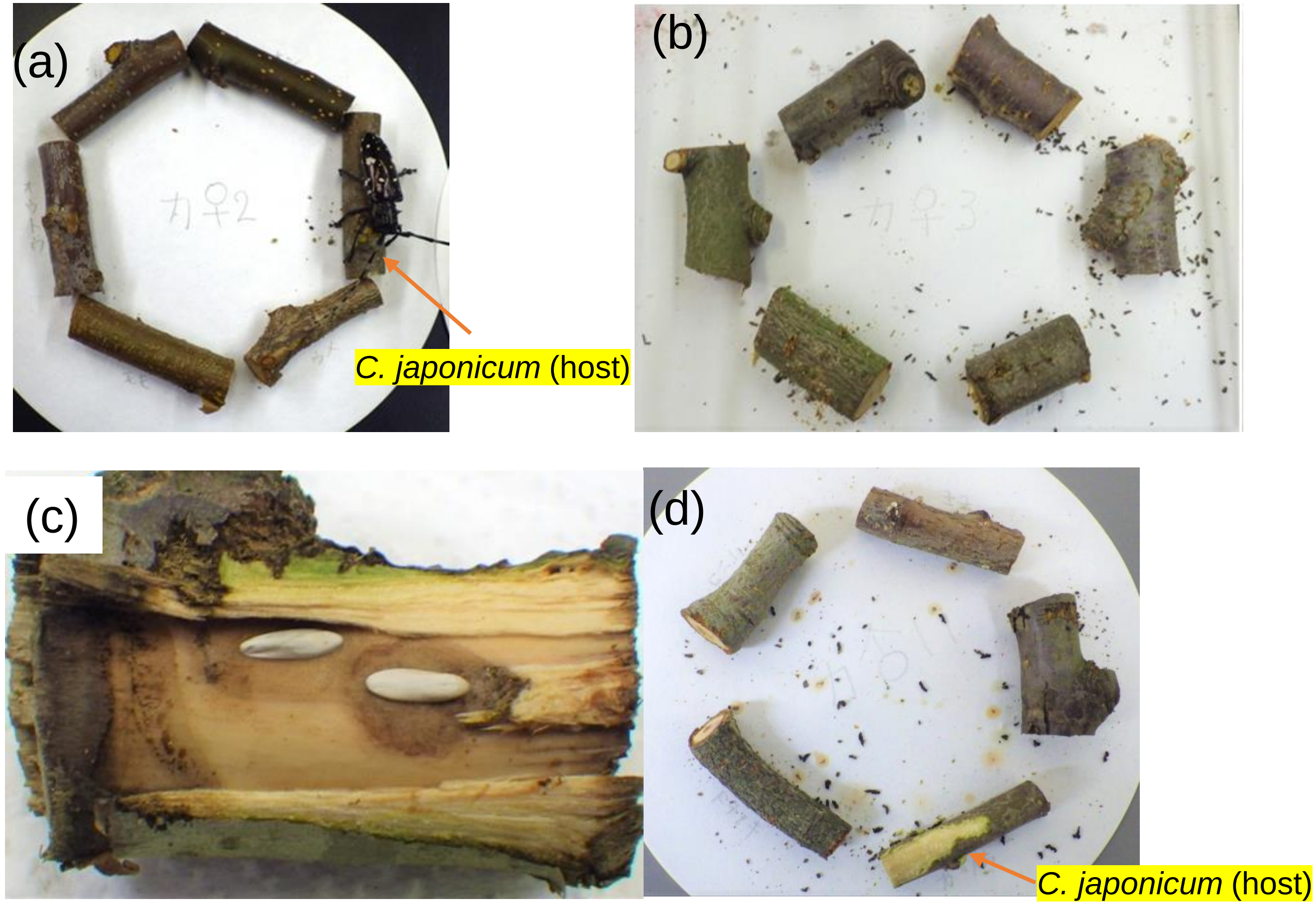

Supplementary figure 1
Photographs of a) feeding-choice assay of a female *Anoplophora glabripennis* among thin branches from one host-plant species and five fruit-tree species; b) oviposition assay after 2 days among thick branches from one host-plant species and five fruit-tree species; c) eggs laid under the bark of a *Pyrus pyrifolia* branch; d) feeding choice assay after 24 h of a male among branches from two host-plant species and three fruit-tree species.

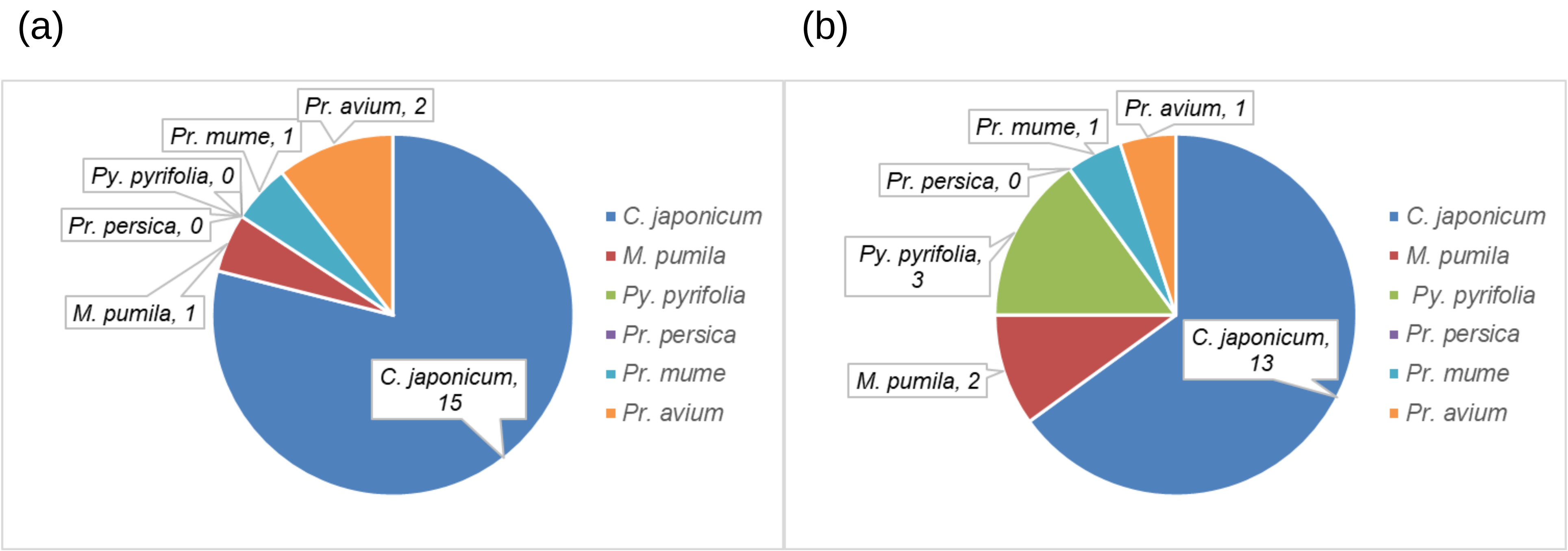

Supplementary figure 2
Feeding-choice assay of adult *Anoplophora glabripennis* beetles among the branches of one host-plant species and five fruit-tree species. a) First bite by males; b) first bite by females. Male: N = 19; female: N = 20.

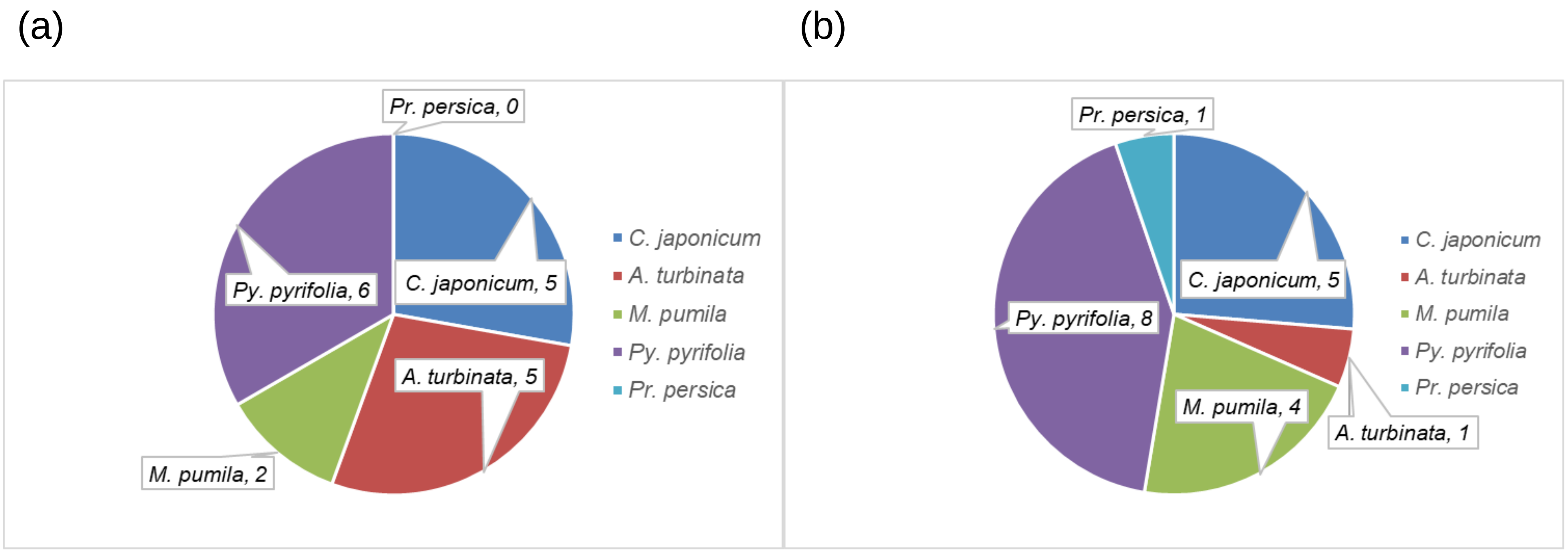

Supplementary figure 3

Feeding-choice assay of adult *Anoplophora glabripennis* beetles among the branches of two host- and three fruit-tree species. a) First bite by males; b) first bite by females. Male: N = 19; female: N = 20.
